# Large-Scale Comparative Genomics of European and Chinese Cattle Breeds Reveals Population Structure, Breeding History, and Adaptive Divergence

**DOI:** 10.3390/ani16091335

**Published:** 2026-04-27

**Authors:** Qiqi Liang, Meng Wang, Jinhua Tang, Hao Liang, Wenjie Han, Fenge Li

**Affiliations:** 1 Key Laboratory of Swine Genetics and Breeding of Ministry of Agriculture and Rural Affairs & Key Laboratory of Agricultural Animal Genetics, Breeding and Reproduction of Ministry of Education, Huazhong Agricultural University, Wuhan 430070, China; liangqiqi@biohuaxing.com (Q.L.); jinhuatang@webmail.hzau.edu.cn (J.T.); h_liang@webmail.hzau.edu.cn (H.L.); 2 Beijing Bio Huaxing Gene Technology Co., Ltd., Beijing 102600, China; wangmeng@biohuaxing.com

**Keywords:** population genomics, genetic diversity, adaptive evolution, selection signatures, deleterious mutation load

## Abstract

Modern cattle comprise two major evolutionary lineages: intensively selected commercial breeds and locally adapted native populations. To investigate their genomic divergence, we performed a comparative population genomic analysis by integrating whole-genome resequencing (WGS) data from multiple representative native breeds and major European commercial breeds. Population genetic analyses showed clear phylogenetic separation between the two groups, with distinct patterns of genetic diversity. Chinese native cattle exhibited generally higher nucleotide diversity (π), lower inbreeding levels, and geographically structured admixed ancestry. Comparative analyses of selection signatures identified 886 candidate selected genes in European commercial breeds, which were primarily enriched in pathways related to production traits, including protein turnover, reproductive regulation, lipid metabolism, and neuro-regulation. In contrast, 50 candidate selected genes in Chinese native cattle were significantly enriched in nervous system functions, particularly ligand-gated ion channel activity and chloride transport (e.g., *GRID2*, *GLRA2/4*, *GABRD*), suggesting neural/ionic regulation may contribute to local adaptation alongside other polygenic mechanisms. Additionally, the two groups also differed in patterns of deleterious mutation load. These findings indicate partially distinct evolutionary trajectories between “production-optimized” and “environment-adapted” cattle and highlight the value of conserving the genetic diversity and adaptive alleles of Chinese native cattle.

## Simple Summary

Chinese native cattle represent a diverse genetic resource that has evolved under localized natural and anthropogenic selection pressures, giving rise to genomic characteristics that are markedly distinct from those of globally commercialized breeds. Through comprehensive genomic analysis, we investigated the contrasting evolutionary trajectories of the native and European commercial cattle breeds. We found that Chinese native cattle maintain higher genetic diversity and a more complex admixed ancestry shaped by geography. Their genomes bear strong selection signals related to nervous system regulation and environmental adaptation, rather than traits for high yield. In contrast, European commercial breeds show selection on coordinated networks for production efficiency but carry a higher burden of potentially harmful mutations. This study provides a genetic basis for conserving the unique adaptive potential of Chinese native cattle and utilizing their resilience genes in future breeding.

## 1. Introduction

The global domestication of cattle is one of the most significant events in the history of agriculture, and its origin can be traced back to Southwest Asia approximately 10,000 years ago [1]. Modern domestic cattle mainly originate from two major subspecies, *Bos taurus* and *Bos indicus*, which are adapted to temperate and tropical environments, respectively [2,3]. With human migration, they spread across the world and, under the combined effects of natural and artificial selection, gave rise to the diverse indigenous breeds and commercial lines observed today [4]. Comparative genomic studies have further shown that convergent selection signals for similar economic traits, such as body size, metabolism, and stress resistance, exist among independently domesticated cattle and even closely related species (e.g., water buffalo), revealing the parallel genetic shaping of key traits during domestication [5]. In this process, Chinese indigenous cattle, as unique genetic resources in East Asia, have played an indispensable role. Through thousands of years of localized domestication and breeding, Chinese indigenous cattle have developed remarkable adaptations to diverse geographic and climatic environments, such as the cold conditions of the Qinghai–Tibetan Plateau, the hot and humid climate of southern China, and the arid regions of northwestern China [6,7,8]. They also retain advantageous traits, including tolerance to low-quality forage, strong stress resistance, and distinctive meat quality, making them valuable genetic resources for addressing future challenges such as climate change, disease threats, and food security.

However, the past half-century of global livestock intensification and standardization, driven by the pursuit of high productivity and efficiency, has seen the rapid expansion of a few commercial breeds (e.g., Holstein, Angus) [9,10]. This trend has precipitated the drastic decline and even endangerment of numerous local breeds worldwide, resulting in an irreversible erosion of genetic diversity. This pattern is equally pronounced in China, posing a severe threat to the conservation of its native cattle genetic resources. A scientific assessment and understanding of the genetic characteristics of these indigenous breeds are therefore prerequisites for formulating effective conservation and sustainable utilization strategies.

In recent years, advances in genomic sequencing have facilitated population genetic studies of Chinese local cattle. Research on individual or regional breeds, such as Hunan, Nanyang, Yanba, and Chaidamu cattle, has begun to unveil their unique genetic backgrounds, demographic histories, and candidate genes associated with adaptation [11,12,13]. Nevertheless, most existing studies are confined to single breeds or specific regions and predominantly rely on single-nucleotide polymorphism (SNP) analysis, lacking a systematic evaluation of other variant types like structural variations (SVs) and copy number variations (CNVs). More importantly, there remains a critical gap: a comprehensive, genome-wide, multi-dimensional comparative study that integrates multiple representative Chinese native breeds with globally dominant commercial breeds. Such a comparison—encompassing population structure, genetic diversity, selection signatures, and deleterious mutation load—is essential for a deeper understanding of the distinct genomic imprints left by two contrasting evolutionary models: “intensive specialized breeding” versus “localized natural-human composite selection”.

To address this gap, we conducted a comprehensive genomic analysis. Utilizing WGS data, we assembled an expanded cohort comprising multiple representative Chinese native breeds and major international commercial breeds. The objectives of this study are threefold: First, to systematically compare the population genetic structure, genetic diversity, linkage disequilibrium (LD) decay patterns, and inbreeding levels between Chinese and European cattle. Second, to identify and contrast the genomic regions and candidate genes under strong selection in each group through selective sweep and functional enrichment analyses, thereby elucidating the divergent selection paradigms: “production-trait oriented” in commercial breeds versus “environmental-adaptation oriented” in native breeds. Third, to assess the deleterious mutation load in both groups, exploring the potential long-term impacts of their distinct breeding histories on genomic integrity. The findings of this study are expected to provide a critical genomic foundation for accurately evaluating the current status of cattle genetic resources in China and for informing scientific in situ and ex situ conservation strategies. Furthermore, the identified selection signatures and key candidate genes will offer valuable insights for the future molecular breeding aimed at harnessing the unique adaptive genetic resources inherent to Chinese native cattle.

## 2. Materials and Methods

### 2.1. Genomic Data Collection and Population Sampling

A total of 111 WGS datasets from global bovine individuals were systematically collected in this study, representing 14 populations that span a wide range of geographical distributions and altitudinal gradients, ensuring a comprehensive assessment of genetic diversity across global cattle breeds. Specifically, the international sample set comprised five European *taurine* breeds: *Hereford*, *Holstein*, *Simmental*, *Angus*, and *Charolais*. The Chinese sample set included eight representative indigenous bovine populations: Mongolian, Chaidamu, Nanyang, Hainan, Yingjing, Pingwu, Zhaojue, and Tibetan cattle. Among all the aforementioned populations, all samples, with the exception of the Hainan cattle, belonged to *Bos taurus*, whereas the Hainan cattle belonged to *Bos indicus*. To construct a robust phylogenetic framework, ten Jinchuan yak *Bos grunniens* individuals were incorporated as outgroups. To ensure transparency and facilitate direct comparison, the number of animals per breed is explicitly stated in Table S1 (ranging from 4 to 15 individuals), reflecting the varying sample sizes across breeds. Detailed sample information, including population name, sampling location, geographic coordinates, altitude, and individual count, is also provided in Table S1. Based on the descriptions provided in the original publications and the associated public project metadata, these datasets were primarily generated for population-scale whole-genome sequencing and population genetic analyses rather than family-based sampling schemes.

### 2.2. Data Preprocessing

Raw sequencing reads underwent quality control and filtering using fastp (v0.23.2) [14]. Default parameters included automatic adapter identification and trimming, removal of low-quality bases (quality score threshold Q20), filtering of reads containing more than 5% ‘N’ bases, and elimination of short sequences below a specified length. High-quality clean reads were then aligned to the reference *Bos taurus* genome (NCBI RefSeq assembly: GCF_002263795.3) using BWA-MEM2 (v2.2.1), with 16 threads employed for parallel computation to enhance processing speed [15,16]. Subsequently, the resulting alignments were processed using samtools (v1.17) for coordinate-based sorting, conversion to BAM format, index generation, and alignment statistics calculation [17]. Finally, PCR duplicates were identified and removed using sambamba markdup (v1.0.1) with the remove duplicates option (-r), yielding high-quality, deduplicated BAM files suitable for downstream variant calling and other genomic analyses [18].

### 2.3. Genome-Wide Variant Discovery: SNP, InDel, SV, and CNV Analysis

Single-sample variant calling was performed on each deduplicated BAM file using the GATK HaplotypeCaller (v4.5.0.0) [19,20]. This process utilized the reference genome (GCF_002263795.3) as a template and generated intermediate gVCF (genomic VCF) outputs. Local-pair Hidden Markov Model parallel computation was enabled with 4 threads to optimize performance. A “scatter-gather” strategy was implemented by dividing the genome into intervals to enhance computational efficiency. Subsequently, all per-sample gVCFs for identical genomic intervals were merged using GATK CombineGVCFs. The merged gVCFs were then subjected to joint genotyping across all samples using GATK GenotypeGVCFs, producing a single, comprehensive VCF file containing genotype information for all individuals. Standard annotations were applied to annotate the functional context of the variants. The resulting VCF files from each genomic interval were then concatenated into a single, cohort-level variant dataset using GATK MergeVcfs. Finally, GATK SelectVariants was employed to extract SNPs and InDels into separate VCF files for downstream analyses. A stringent hard-filtering protocol was applied to the raw SNPs using GATK VariantFiltration with the following criteria: QD < 2.0, MQ < 40.0, QUAL < 30.0, SOR > 3.0, MQRankSum < −12.5. A relatively relaxed filtering standard was applied to the InDel dataset. High-quality SNPs and InDels variant datasets were ultimately obtained.

Building upon the small variant discovery, we further conducted a systematic analysis of SVs across the entire genome. First, structural variant calling was performed on all deduplicated BAM files using smoove (v0.2.8) [21]. This tool integrates the functionalities of LUMPY and SVTyper, enabling the systematic detection of various types of SVs, including deletions, duplications, inversions, and translocations. Subsequently, the individual VCF files generated by smoove were consolidated into a single, high-quality cohort-level SV dataset using SURVIVOR. To systematically identify copy number variations, we employed CNVnator (v0.8.2) to detect copy number gains and losses across the whole genome by analyzing sequencing depth fluctuations within fixed-size windows (default 100 bp) [22]. The genomic regions were subsequently partitioned using statistical models to identify CNVs with significant depth differences, which were then output in standardized VCF format. The independently detected CNV results from all samples were integrated and merged using the SURVIVOR software (v1.0.7), generating a unified, comparable population-level CNV dataset [23].

### 2.4. Population Genetic Structure and Diversity Analysis

To characterize population genetic structure, a genetic relationship matrix (GRM) was computed using GCTA (v1.24.2) [24,25], followed by Principal Component Analysis (PCA) [26]. Concurrently, the software ADMIXTURE (v1.3.0) was employed to estimate ancestral population contributions [27]. The optimal number of clusters (K) was determined by cross-validation error to reveal patterns of genetic admixture among populations. To elucidate evolutionary relationships, a Neighbor-Joining phylogenetic tree was constructed from the high-quality, filtered SNP dataset using the TreeBest package (v1.9.2). The tree’s topology was evaluated through 1000 bootstrap resampling iterations. The resulting phylogeny was visualized using an R script to corroborate the findings from the population structure analyses [28,29].

To further investigate population-specific genetic characteristics, a series of downstream analyses were performed on the quality-controlled SNP dataset. The decay pattern of LD within predefined populations was assessed using PopLDdecay (v3.41) [30,31]. Runs of Homozygosity (ROH), indicative of inbreeding or founder effects, were identified across the genomes of 101 samples (excluding yak outgroups) using PLINK (v1.90) [32] and VCFtools (0.1.16) [33] via the PLINK --homozyg module to scan for extended homozygous segments. Population genetic diversity was quantified by calculating the sliding-window nucleotide diversity (π) using the VCFtools --window-pi module. Individual inbreeding coefficients (F) were computed using PLINK --het (v1.90) based on observed and expected heterozygosities, reflecting the deviation of homozygote counts from Hardy-Weinberg equilibrium expectations [34,35].

### 2.5. Inter-Population Genetic Differentiation and Selection Signature Detection

To assess the degree of genetic differentiation between populations, the fixation index (*F*_ST_) [36] was calculated using the --weir-fst-pop function in VCFtools (v0.1.16). Subsequently, the sequence diversity statistics (π) was calculated for each population using the --window-pi module in VCFtools with a 40 kb window sliding in 20 kb steps [37]. Finally, to conduct a comprehensive scan for selection signatures, genomic regions corresponding to the highest *F*_ST_ values (top 5%) were overlapped with those harboring the lowest nucleotide diversity (π, bottom 5%). The intersecting regions were defined as candidate genomic loci under strong directional selection.

To decipher the potential biological functions of candidate genes within these strongly selected regions, systematic Gene Ontology (GO) [38] and Kyoto Encyclopedia of Genes and Genomes (KEGG) [39] pathway enrichment analyses were performed using the clusterProfiler package (v4.0) in the R environment [40,41,42].

### 2.6. Deleterious Mutation Load Analysis

To quantify and compare the potential burden of deleterious mutations across different genetic populations, variant annotation and classification were first performed using snpEff (v5.2-3) [43]. Subsequently, key mutational load metrics were calculated for each individual sample, including the count of Loss-of-Function (LoF) variants, Missense variants, Synonymous variants, and the proportion of Damaging variants relative to the total number of variants. Finally, R scripts were utilized to perform statistical comparisons and generate visualizations of these metrics across the different populations.

All statistical analyses were performed using the R statistical computing environment [44]. Key packages included ggplot2 for visualization [45], dplyr for data manipulation [46], and clusterProfiler for functional enrichment analysis [40]. Interactive visualization was conducted using plotly [47]. Additional R packages such as RColorBrewer [48], ggforce [49], scales [50], getopt [51], argparse [52], gtools [45], and tidyverse [53] were used to support data processing and graphical presentation.

## 3. Results

### 3.1. Quality Evaluation of Whole-Genome Resequencing Data

This study performed a comprehensive analysis of published WGS data from 111 cattle samples representing diverse global breeds (Table S2), aiming to construct a panoramic map of genetic variation within the genus *Bos*. To ensure data integrity, we implemented stringent quality control on all raw sequencing data (Table S3). The results demonstrate that all samples met high-quality standards: an average of 39.84 Gb of raw sequence data per individual, an average effective mapping rate of 99.43%, with 99.3% of bases achieving a Q30 quality score (indicating an error rate < 0.1%), and a mean GC content of 43.91%, confirming the high reliability and accuracy of the datasets. The high-quality clean reads were subsequently aligned to the reference genome, and alignment efficiency along with genomic coverage were evaluated (Table S4). The analysis revealed a mean mapping rate of 99.00%, an average sequencing depth of 14.58×, and a mean coverage of 92.00% of the genome at ≥5× depth. Collectively, these quality metrics validate the robustness of the integrated high-fidelity genomic dataset, providing a solid foundation for subsequent in-depth analyses of population genetic structure, phylogenetic relationships, and adaptive evolutionary divergence among cattle breeds.

### 3.2. Landscape of Genomic Polymorphisms in the Bos Genome

A systematic analysis of the WGS data led to the identification of four major classes of genetic variants in the *Bos* genome. A total of approximately 96.98 million high-confidence SNPs, 10.55 million InDels, 0.27 million SVs, and 0.58 million CNVs were cataloged, constituting a comprehensive map of genomic variation in *Bos* species. Functionally, the distribution of SNPs exhibited a canonical pattern, predominantly localized to intergenic regions (57.25%) and introns (38.13%), reflecting relaxed selective constraints in non-coding areas (Table S5). The observed transition-to-transversion ratio (Ts/Tv) of 2.32 aligns with the established norms for mammalian genomes, further validating the quality of the SNP dataset. The genomic distribution of InDels followed a similar pattern, primarily occurring in intronic (39.16%) and intergenic regions (56.82%) (Table S6). Both SVs and CNVs were widely distributed across the genome. SVs were largely dominated by large fragment deletions (45.21%), while CNVs showed a relatively balanced distribution between deletion and duplication classes (Tables S7 and S8).

**Figure 1:**
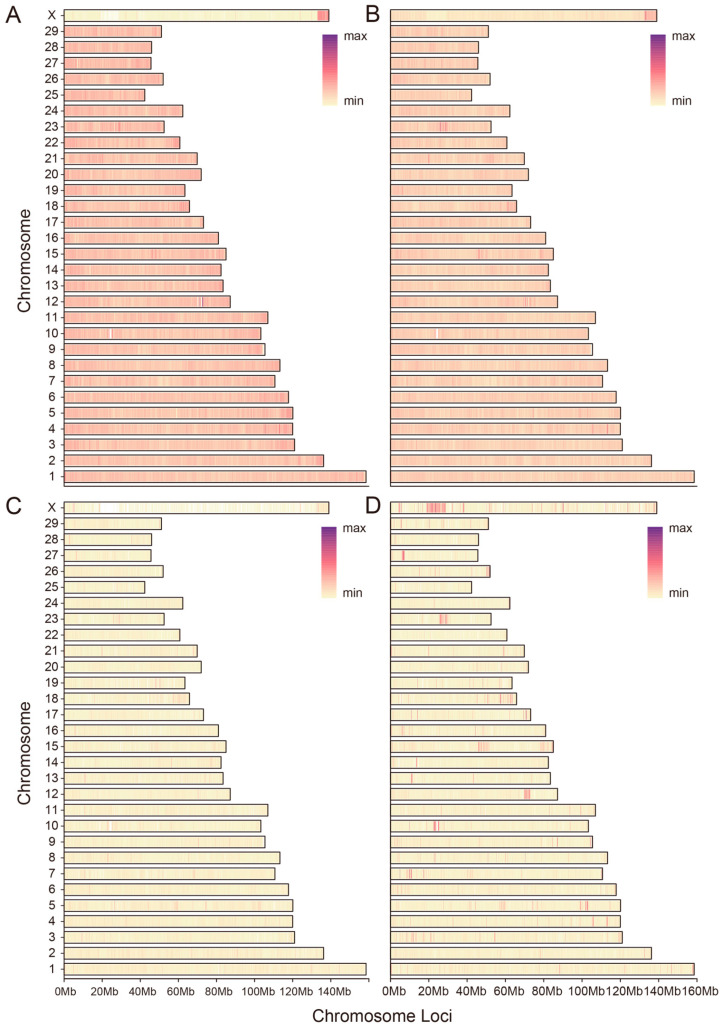
Chromosomal Distribution Landscape of Genome-wide Variants in Cattle Populations. (**A**) SNP density profile along the genome, showing relatively continuous distribution across autosomes and reduced density on the X chromosome; (**B**) InDel density profile, exhibiting a pattern similar to SNPs; (**C**) Structural variation (SV) density profile; (**D**) Copy number variation (CNV) density profile.

Furthermore, Figure 1 illustrates the chromosomal density distribution of the four variant types across the bovine genome. All variant classes exhibited a non-uniform distribution along chromosomes. SNPs (Figure 1A) and InDels (Figure 1B) showed relatively continuous patterns along most autosomes but were sparser on the X chromosome. In contrast, SVs (Figure 1C) and CNVs (Figure 1D) displayed a more heterogeneous distribution, characterized by distinct hotspots, suggesting potential regions of elevated structural rearrangement activity or replication instability during evolution.

### 3.3. Population Genetic Structure Analysis

**Figure 2:**
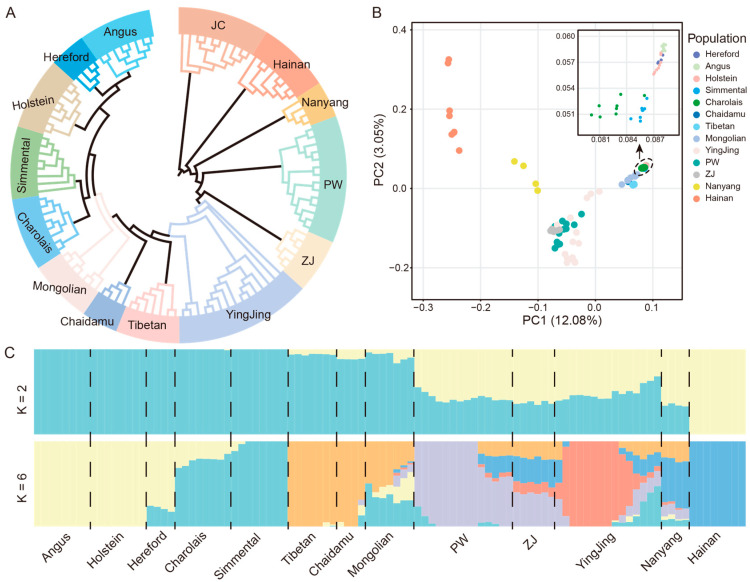
Population Genetic Structure of Chinese Indigenous and Foreign Cattle Based on Genome-wide SNP Data. (**A**) Phylogenetic tree constructed from 111 cattle samples to visualize genetic relationships among populations; (**B**) Principal component analysis (PCA) plot showing the genetic differentiation pattern among the 14 cattle populations; (**C**) Population structure inferred by STRUCTURE at K = 2, 6. Each color represents a distinct ancestral genetic component, and the proportion of colors in each vertical bar indicates the percentage of different ancestral components in that individual’s genome.

Based on genome-wide SNP data, we performed population genetic structure analyses of the 111 samples. The phylogenetic tree (Figure 2A) revealed a clear dichotomy: cattle from native and abroad clustered distinctly on either side. Five European breeds—Angus, Holstein, Charolais, Simmental, and Hereford—formed a tight outgroup (Foreign), while all Chinese native breeds clustered into another major branch (Native). Furthermore, Mongolian cattle occupy an intermediate position between Chinese and European cattle. The samples for this population are derived from Mongolian cattle in both Mongolia and domestic China. This indicates significant genetic divergence between foreign and Chinese native cattle breeds. Principal component analysis (PCA) further revealed this differentiation pattern (Figure S1). The first principal component (PC1, 12.08%) clearly separated foreign purebreds from most Chinese native breeds, with the latter exhibiting a wider range of genetic variation along PC1. Figure 2B details the clustering of the 13 populations, where some native breeds show a certain degree of overlap with European breeds along PC1, suggesting potential historical introgression events.

The STRUCTURE analysis results show that at K = 2, all individuals were divided into two main ancestral components: one representing foreign European ancestry and the other representing Chinese native ancestry (Figure 2C). As K increased, the population structure became increasingly refined. At K = 3, the native and European groups were clearly separated. At K = 4, internal differentiation began to appear within Chinese native breeds, with southwestern breeds beginning to separate from plateau types in western China; Huang-Huai-Hai plain types also showed distinct genetic compositions from Hainan cattle, attributable to differential *Bos indicus* introgression, with Nanyang cattle retaining notable *Bos indicus* influence alongside predominant *Bos taurus* ancestry. By K = 6, the genetic structure of each native population became clearer, forming several distinct genetic clusters, including Qinghai-Tibetan plateau, southwestern mountainous, Huang-Huai-Hai plain, and South China coastal types, reflecting their complex admixed origins and regional adaptive evolution. Figure S2 shows the STRUCTURE analysis results for K = 3, K = 4, and K = 5, visually presenting the dynamic process of population differentiation at different K values.

Collectively, these observations are consistent with Chinese native cattle being genetically differentiated from European purebred cattle and displaying a complex admixed structure—patterns that may reflect, at least in part, the documented taurine-indicine admixture alongside long-standing geographic and breeding histories.

### 3.4. Comparative Analysis of Population Genetic Characteristics

**Figure 3:**
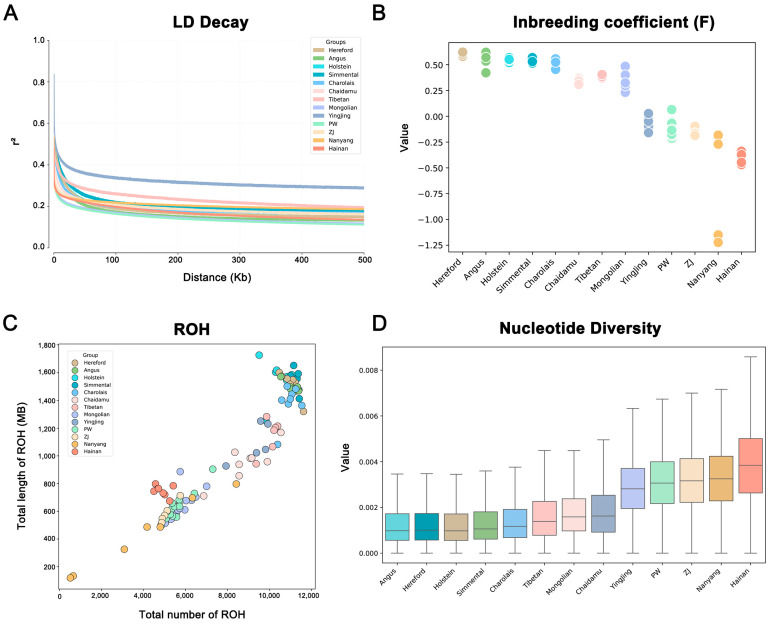
Population Genetic Diversity and Inbreeding Related Metrics in Cattle. (**A**) Linkage disequilibrium (LD) decay plot, showing the extent of LD (measured as r^2^) as a function of genetic distance (in kb) across different cattle populations, with each line representing a population; (**B**) Distribution of genomic inbreeding coefficients (F) among the studied cattle populations; (**C**) Relationship between the total number of runs of homozygosity (ROH) and the total length of ROH for individuals across populations; (**D**) Nucleotide diversity (π) across the 13 cattle populations.

Figure 3 compares the genetic characteristics of Chinese native and foreign cattle breeds across four aspects: LD, F value, ROH, and nucleotide diversity (π). LD decay analysis (Figure 3A) shows that most foreign purebreds have similar decay rates to Chinese natives, while Hereford and Chaidamu exhibit the slowest decay, suggesting smaller effective population sizes or strong selection. The inbreeding coefficient analysis (Figure 3B) indicates that foreign purebreds generally have higher F values than Chinese natives, reflecting higher inbreeding levels. ROH analysis (Figure 3C) further confirms this, revealing more and longer homozygous fragments in foreign breeds. Conversely, nucleotide diversity analysis (Figure 3D, Figure S3) shows that foreign breeds generally have lower π values than Chinese natives, indicating reduced genetic diversity. Collectively, these results reveal contrasting genetic backgrounds: foreign purebreds have undergone intensive artificial selection, leading to high inbreeding and low diversity, whereas Chinese native cattle retain higher genetic diversity and lower inbreeding levels.

### 3.5. Functional Divergence of Candidate Selected Genes

**Figure 4:**
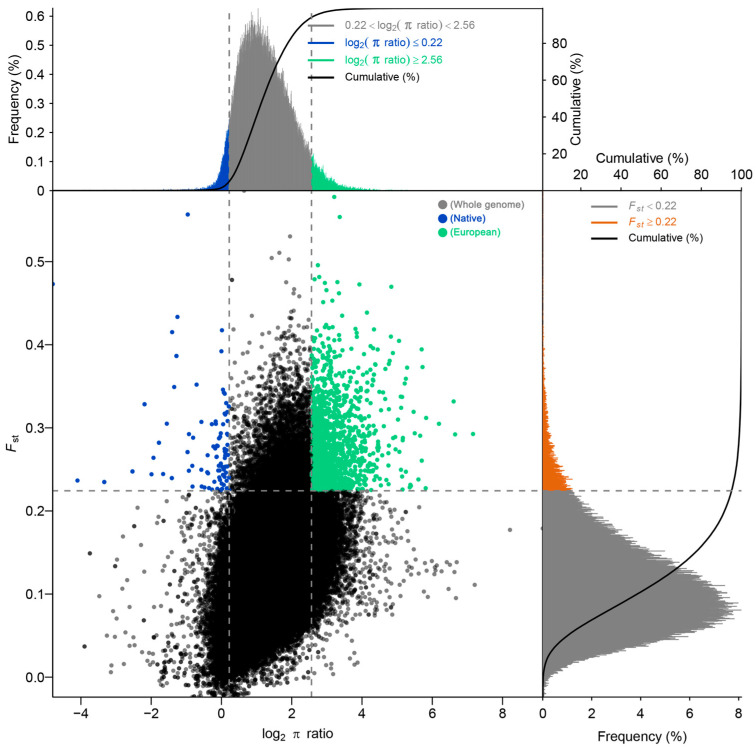
Selective Sweep Analysis Identifies Divergent CSGs Between Native and European Cattle Populations. Blue dots represent CSGs under positive selection in the Native population; green dots represent CSGs under positive selection in the European population. Dashed lines indicate genome-wide significance thresholds for selection signals.

To investigate the divergent selective pressures acting on native and European cattle breeds during their prolonged independent evolution, we performed selective sweep analyses comparing the Native together with Mongolian cattle and European breeds (Figure 4). The analysis revealed a significant functional differentiation in the genes targeted by selection between the two groups (Tables S9 and S10). In the European cattle populations, 886 candidate selected genes (CSGs) were significantly enriched in multiple core pathways associated with production performance and physiological regulation. These included protein turnover and degradation (involving ubiquitin ligase genes such as *BIRC7*, *BTRC*, *CDC34*); growth and reproductive regulatory networks, in which *IGF1* plays a central role within the growth hormone/IGF axis. Previous studies have shown that variation in *IGF1* and its downstream signaling is associated with growth performance and reproductive traits in livestock, including body weight, average daily gain, and fertility-related traits, partly mediated through circulating *IGF-1* levels [54,55,56]; lipid metabolism (e.g., *FABP4*), affecting meat quality and energy storage; neurological regulation (e.g., GABA receptor subunits GABRA3 and GABRB1), which are involved in inhibitory neurotransmission and have been implicated in the regulation of stress responses and aggressive behavior, and may contribute to behavioral adaptations during domestication [57,58]; and immune regulation (e.g., *IRAK3*, *STAT6*), crucial for health maintenance.

In contrast, the functional core of the 50 CSGs identified in Native together with Mongolian cattle was highly focused on environmental adaptation and fundamental physiological regulation. Pathways related to nervous system ligand-receptor interactions and ion channel functions were significantly enriched, with key genes including the glutamate receptor *GRID2*, glycine receptors *GLRA2/GLRA4*, and the GABA receptor *GABRD*. These genes are involved in neurotransmission and the regulation of neuronal excitability, particularly through inhibitory signaling and chloride ion homeostasis, which are fundamental mechanisms underlying neural stability and stress responsiveness [59]. Such processes may contribute to the ability of native cattle populations to cope with variable and challenging environmental conditions. Consistent with our findings, population genomic studies in Chinese indigenous cattle, such as Bashan cattle, have also identified signatures of selection in genes related to neural function and environmental adaptation [60]. Furthermore, the enrichment of genes involved in nutritional metabolism (*LCT*, *MCM6*), cell cycle regulation (*CCNB3*), deubiquitination (*USP9X*), and potential disease resistance (*IL1RAPL2*, *ABCB5*) further reflects coordinated selection on fundamental physiological systems contributing to organismal fitness. Consistent with these observations, population genomic studies in Chinese indigenous cattle have reported similar patterns of selection. For instance, Bashan cattle show signatures of selection in genes associated with neural regulation and environmental adaptation [60], while Xiangnan cattle and Hetian cattle exhibit enrichment of immune response, metabolic regulation, and cellular homeostasis pathways [6,61]. These consistent findings across multiple native cattle breeds suggest that survival, stress resistance, and physiological homeostasis under complex environmental conditions are key drivers of selection in indigenous cattle populations.

The unique selective pressures experienced by native breeds to maintain survival, resist adversity, and preserve basic physiological homeostasis in diverse natural habitats. The functional enrichment results for the selected genes in both domestic and European cattle are detailed in Figures S4–S7.

### 3.6. Comparative Analysis of Deleterious Mutation Load

**Figure 5:**
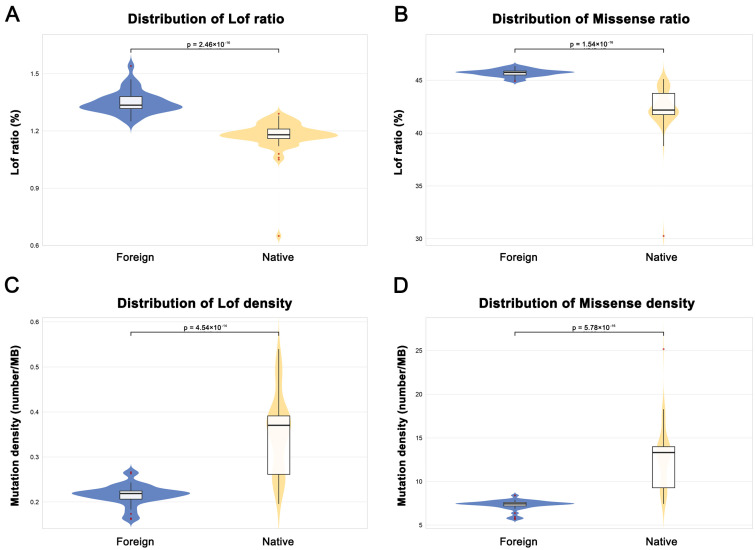
Deleterious Mutation Burden in European Versus Native Cattle Populations. (**A**) Proportion of Loss-of-Function (LoF) mutations in European versus native cattle; (**B**) Proportion of missense mutations in European versus native cattle; (**C**) Genomic density of Loss-of-Function (LoF) mutations in European versus native cattle; (**D**) Genomic density of missense mutations in European versus native cattle.

To further understand the genetic background differences between the native and European cattle breeds, we quantified the deleterious mutation load across different populations. Our results indicate that European purebred cattle, subjected to long-term intensive artificial selection, have accumulated significantly higher proportions of deleterious mutations, particularly Loss-of-Function (LoF) and missense variants, compared with the native breeds (Figure 5A,B). Paradoxically, however, the genomic density of these deleterious mutations is significantly lower in European breeds than in the native ones (Figure 5C,D). This distinctive “high-proportion, low-density” pattern likely reflects the evolutionary history of European breeds, where, under small effective population sizes (e.g., genetic bottlenecks), selection for a few high-production traits may have tolerated elevated local proportions of deleterious variants within specific genomic regions, while limiting overall genome-wide accumulation of deleterious load.

In contrast, native breeds exhibit a distinct pattern characterized by lower overall proportions of putatively deleterious variants but higher genomic density across the genome. This pattern may reflect differences in demographic history, selection intensity, and long-term environmental adaptation. Rather than indicating a directional reduction in deleterious mutations, it likely represents a complex balance between genetic drift and selection acting on standing genetic variation. Consequently, these populations may retain substantial genetic diversity while showing distinct distributions of potentially deleterious variants, reflecting heterogeneous evolutionary and breeding histories.

## 4. Discussion

This integrative genomic analysis reveals profound genetic divergence between the native and European commercial cattle breeds, manifesting as systematic differences in population structure, diversity profiles, selection signatures, and mutational load patterns. This underscores their distinct evolutionary trajectories and provides a new molecular framework for understanding, conserving, and utilizing native genetic resources.

In contrast to genetically homogenized and highly “purified” European commercial breeds, ADMIXTURE and phylogenetic analyses reveal that native cattle constitute a classic admixed population, whose genomes bear the signature of complex ancestral hybridization, consistent with previous whole-genome studies of Chinese indigenous cattle [2,62]. The geographically correlated genetic clusters observed at K = 6 (e.g., Plateau, southwestern mountainous) indicate that their evolutionary trajectory is not a simple linear divergence, but rather the outcome of multiple, temporally and spatially distinct admixture events between local ancestral populations and introduced *Bos taurus* and *Bos indicus* lineages. This nuclear genomic pattern of admixture is further supported by matrilineal evidence. Mitochondrial DNA (mtDNA) phylogenetic studies suggest that taurine cattle migrated from the north and indicine cattle from the south into central China, with taurine populations persisting in the northwestern fringes and undergoing a demographic expansion in the Longdong region of Gansu Province, followed by a northward migration of indicine cattle [62,63]. This proposed maternal demographic history is congruent with the autosomal admixture pattern elucidated in the present study. Moreover, recent pangenome analyses have further revealed that Chinese indicine cattle harbor substantial genomic components introgressed from multiple *Bos* species, contributing to their elevated genetic diversity and complex genomic architecture [64]. A similar admixed genetic architecture is prevalent among other indigenous Chinese cattle breeds, as demonstrated in studies of Hunan and Hubei cattle [11,12].

Furthermore, the PCA results of this study indicate genetic overlap between some indigenous breeds and European breeds, which is corroborated by previous evidence of introgression from European South Devon and Red Angus cattle observed in Pingliang red cattle [65]. These findings collectively suggest that historical gene flow between Chinese and European cattles was not a sporadic event in the formation of Chinese native cattle populations. This sustained gene flow, coupled with local complex eco-geographic isolation, has shaped the “admixed yet structured” population genetic architecture of Chinese native cattle. This pattern is not only a key historical driver of their high genetic diversity but has also provided critical genetic raw material—through the introduction and recombination of diverse adaptive alleles—for their successful radiation and local adaptation across heterogeneous ecological environments.

This study confirms that native cattle populations generally possess significantly higher nucleotide diversity (π) and lower inbreeding levels than European cattles. High genetic diversity implies a richer reservoir of potentially adaptive alleles, serving as a key “genetic buffer” against unpredictable selective pressures such as climate change and emerging diseases. Meanwhile, low inbreeding levels are directly associated with reduced risks of inbreeding depression, favoring the long-term viability and reproductive health of populations.

These findings are highly consistent with genomic studies of native cattle breeds across various regions of China, collectively underscoring the widespread characteristic of Chinese native cattle as a high-diversity genetic resource pool. For instance, Xiangxi cattle exhibit higher genomic diversity and weaker signals of artificial selection compared with commercial breeds [66]; genetic diversity assessments of the Qinchuan cattle conservation population reveal relatively abundant genetic variation (Ho = 0.275) [67]; and the Xinjiang Brown cattle population also demonstrates high genetic diversity (He = 0.376) [68]. Similar to the native cattle populations in this study, these breeds maintain high heterozygosity and low inbreeding loads. In contrast, the trait optimization achieved in European commercial breeds through intensive breeding has come at the cost of reduced genetic diversity and accumulated inbreeding load. For example, pedigree evaluations of Ecuadorian Holstein–Friesian cattle indicate that the combined use of artificial insemination, embryo transfer, and genomic selection has led to the most significant loss of genetic diversity [69]. This case aligns closely with the generally lower π values and higher inbreeding coefficients observed in European commercial breeds in this study. Such narrowing of the genetic base poses potential risks to their long-term adaptability and sustainability, potentially leaving them without the necessary genetic buffer to face new environmental or disease challenges.

Therefore, the native cattle populations constitute a more valuable and highly resilient “living gene bank” than European cattles. This resource is not only crucial for the current conservation of local breeds and the development of distinctive traits but also represents a strategic genetic reserve for addressing future global challenges such as climate change, epidemic diseases, and food security. Notably, selective sweep analyses suggest potential functional divergence between breed groups. In European commercial breeds, selection signals tend to overlap with loci associated with economically important traits such as growth and production performance (e.g., meat, milk, and reproductive traits) [70,71]. In contrast, native breeds show enrichment of selection signals in genes involved in neural system-related processes, including *GRID2*, *GLRA2/GLRA4*, and *GABRD*. Previous studies of selective sweeps in domesticated animals have also reported that genes under selection frequently involve neurobehavioral functions and sensory or neural regulation pathways [70,72]. This pattern raises the hypothesis that modulation of neuroregulatory pathways may have potentially contributed to local environmental adaptation; however, the precise functional consequences remain to be experimentally validated. Enrichment of genes like *LCT/MCM6* and *IL1RAPL2* further highlights local adaptation in nutrition and immunity [73,74,75]. Enrichment of genes such as *LCT/MCM6* and *IL1RAPL2* further highlights signatures of local adaptation related to nutrition and immune function. These findings are consistent with a model in which the genomes of native breeds have been shaped not only by modest artificial selection for production traits but also by sustained environmental pressures that maintain genetic variants contributing to resilience.

Furthermore, the pattern of deleterious mutations identified in this study provides an additional layer of evidence supporting the genetic divergence discussed above. The “high proportion, low density” pattern observed in European breeds likely represents a genomic cost incurred under the joint effects of a small effective population size and intense artificial selection. Under this scenario, selective sweeps aimed at rapidly fixing a limited number of favorable alleles associated with high production traits can increase the frequency of beneficial alleles in target genomic regions. Simultaneously, however, tightly linked deleterious variants may be co-fixed via genetic hitchhiking, leading to an elevated local proportion of deleterious mutations. Owing to the small effective population size and strong genetic drift, however, these deleterious mutations may be distributed more sparsely across the genome, manifesting as a lower genomic-wide density. This finding is consistent with observations in Ganxi goats, where highly inbred breeds show an enrichment of deleterious homozygous genotypes within long ROH, underscoring that the accumulation of deleterious mutations is a common genetic risk in populations subjected to small size and strong directional selection [76].

In contrast, the native cattle exhibit the opposite “low proportion, high density” pattern. This likely reflects a different genomic purging mechanism operating under a history of larger effective population sizes and a composite natural-human selection regime centered on adaptation. This pattern bears similarity to findings in Guangfeng goats, which also display high genetic variability and a comparable clustering of deleterious alleles, both reflecting distinct genomic “tolerance” strategies under relatively natural selection pressures [76]. Notably, a study of mitochondrial genomes across 18 global cattle breeds also revealed a more than five-fold difference in deleterious mutation load among breeds, with younger breeds exhibiting a higher proportion of deleterious SNPs compared with older breeds [77]. This finding further corroborates the general principle that breed history and population size profoundly influence the accumulation of deleterious mutations.

The findings of this study provide direct guidance for the scientific management of the genetic resources of Chinese native cattle. Conservation strategies should extend beyond the simple “maintenance of breeds” to actively and dynamically preserve the genetic diversity of their core populations and their unique adaptive allele spectrum. Indiscriminate introduction of European bloodlines may lead to short-term improvements but could also compromise locally adapted genetic variation. Therefore, greater emphasis should be placed on the conservation and utilization of indigenous adapted breeds, which harbor valuable genetic resources shaped by long-term environmental pressures. Through controlled crossbreeding and selective breeding strategies, new breeds that combine high productivity, superior quality, and strong stress resilience can be developed.

At the same time, this study has certain limitations. For example, sample sizes (ranging from 4 to 15 individuals) for some rare local breeds remain limited. This sample size imbalance may reduce the power to detect low-frequency variants and rare structural events and may increase sampling variance in estimates of nucleotide diversity and inbreeding metrics in small populations. While major population structure patterns (e.g., European versus Chinese cattle) are robust, finer-scale structure and admixture signals in breeds with limited sample sizes should be interpreted with caution. Similarly, results from selection scans and downstream functional enrichment analyses should be interpreted as indicative of broad biological trends rather than exhaustive gene sets, as rare signals may be underrepresented. Moreover, the lack of detailed physiological and behavioral phenotypic data directly linked to key candidate genes restricts the mechanistic interpretation linking “genotype” to “phenotype”. Future research should expand sample sizes to enable more refined population genetic analyses and integrate multi-omics approaches to construct “genotype–phenotype–environment” association networks. Such efforts will contribute solutions derived from Eastern indigenous genetic resources to address the sustainability challenges facing global animal husbandry.

## 5. Conclusions

Chinese native cattle represent a valuable genetic resource characterized by relatively high genetic diversity, a complex admixed genetic background, and considerable adaptive potential. Selection sweep analyses have identified a set of candidate genes that may be associated with environmental adaptation and economically important traits, providing preliminary insights into their genetic basis. However, given the uneven sample sizes across breeds and the inherently polygenic nature of these traits, the results should be interpreted with caution. The identified genes likely represent only a subset of the underlying genetic architecture rather than a comprehensive representation of the molecular mechanisms. Overall, this study provides a useful genomic resource and a foundation for future investigations. Further studies incorporating larger sample sizes and integrative multi-omics and phenotypic data will be essential to better elucidate genotype–phenotype relationships and to support conservation and breeding efforts in native cattle.

## Data Availability

All sequencing data analyzed in this study were obtained from publicly available resources. The datasets are deposited in the National Center for Biotechnology Information (NCBI) Sequence Read Archive (SRA) under the following BioProject accession numbers: PRJNA176557, PRJNA379859, PRJNA737584, PRJNA598339, PRJNA379860, PRJNA396672, PRJNA1369724, PRJNA256210, PRJNA343262, and PRJNA483376. These publicly accessible datasets support reproducibility and further research in cattle genomics.

## Supplementary Materials

The following supporting information can be downloaded at: https://www.mdpi.com/article/10.3390/ani16091335/s1, Figure S1: PCA Analysis of Genetic Differentiation Between Native and European Cattle Populations; Figure S2: STRUCTURE Analysis of Cattle Populations at Different Ancestral Component Levels (K = 3, 4, 5).; Figure S3: Nucleotide Diversity Comparison Between Native and European Cattle Groups; Figure S4: GO Enrichment Analysis of Positively Selected Genes in Native Indigenous Cattle; Figure S5: KEGG Enrichment Analysis of Positively Selected Genes In Native Indigenous Cattle; Figure S6: GO Enrichment Analysis of Positively Selected Genes In European Cattle; Figure S7: KEGG Enrichment Analysis of Positively Selected Genes in European Cattle; Table S1: Regional Distribution and Sampling Information of Selected Cattle Populations; Table S2: Characteristics of Cattle Samples Included in the Whole-Genome Resequencing Study; Table S3: Quality Control Metrics of Raw Whole-Genome Resequencing Data; Table S4: Summary of Whole-Genome Resequencing Alignment Metrics And Depth of Coverage By Sample; Table S5: Annotation Statistics of Snps Identified From Whole-Genome Resequencing Analysis; Table S6: Genomic Annotation And Distribution of Insertion and Deletion (Indel) Variants; Table S7: Classification and Distribution of Structural Variants (Svs) Across Genomic Regions; Table S8: Functional Annotation and Classification Summary of Genome-Wide Copy Number Variants (Cnvs); Table S9: Comparative GO Enrichment (MF, *P* < 0.05) of Selection Sweep Genes in European and the Other Native Breeds; Table S10: Comparative KEGG Enrichment (*P* < 0.05) of Selection Sweep Genes in European and the Other Native Breeds.

## Abbreviations

WGS	Whole-genome resequencing
Π	Nucleotide diversity
SNP	Single nucleotide polymorphism
SVs	Structural variations
CNVs	Copy number variations
LD	Linkage disequilibrium
gVCF	Genomic VCF
GRM	Genetic relationship matrix
PCA	Principal Component Analysis
ROH	Homozygosity
F	Individual inbreeding coefficients
*F*_ST_	Fixation index
GO	Gene Ontology
KEGG	Kyoto Encyclopedia of Genes and Genomes
LOF	Loss-of-Function
CSGs	Candidate selected genes
