# Supplementary material for: Large-Scale Comparative Genomics of European and Chinese Cattle Breeds Reveals Population Structure, Breeding History, and Adaptive Divergence
Source: Animals (Basel). 2026 Apr 27;16(9):1335. doi: 10.3390/ani16091335 (PMC13162682; doi:10.3390/ani16091335)
Supplement: Supplementary file 1 [file animals-16-01335-s001.zip › supplementary file Figures.pdf]

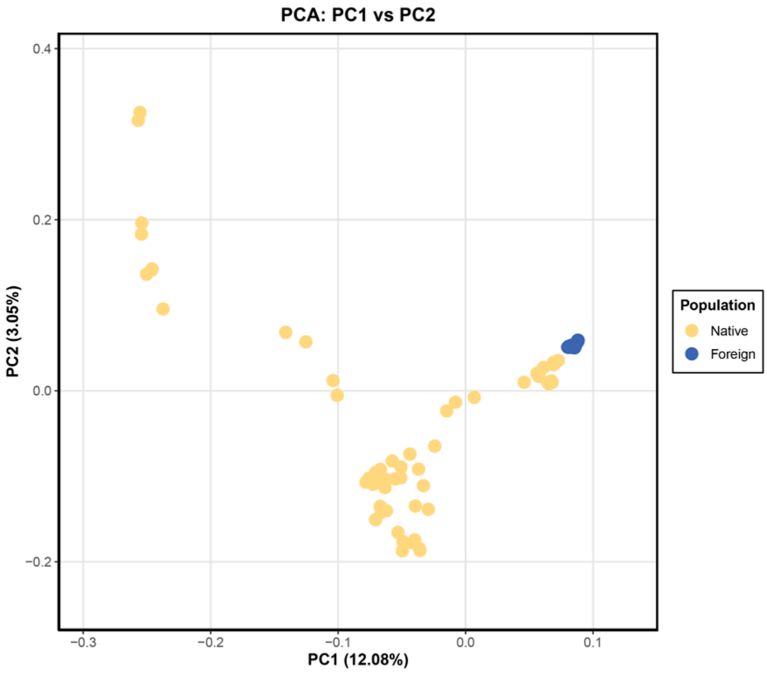


**Figure S1.** PCA Analysis of Genetic Differentiation Between Native and European Cattle Populations.

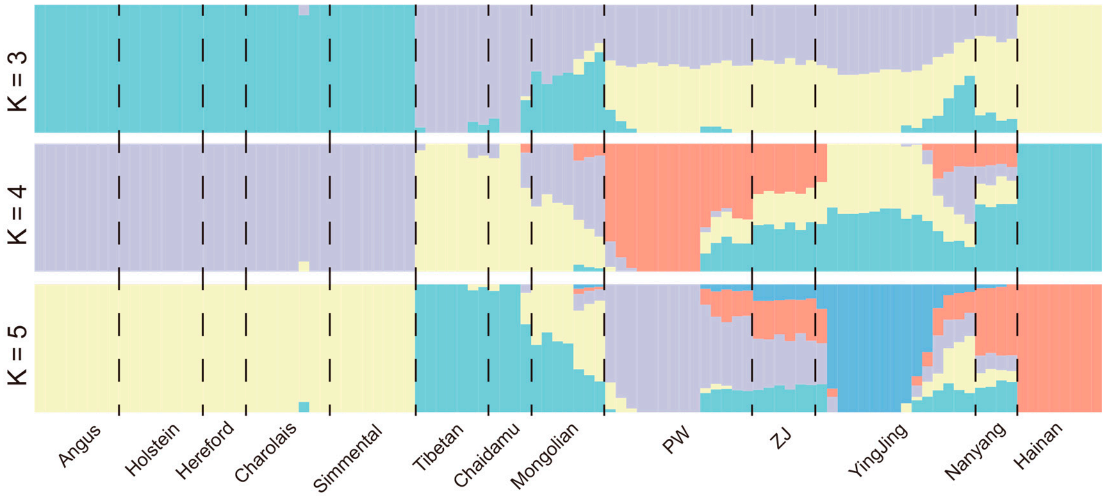


**Figure S2.** STRUCTURE Analysis of Cattle Populations at Different Ancestral Component Levels (K = 3, 4, 5).

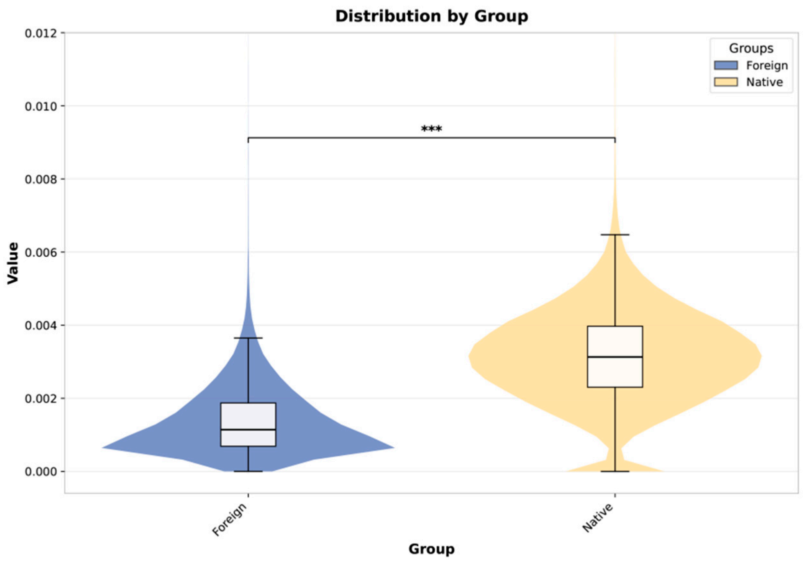


**Figure S3.** Nucleotide Diversity Comparison Between Native and European Cattle Groups.

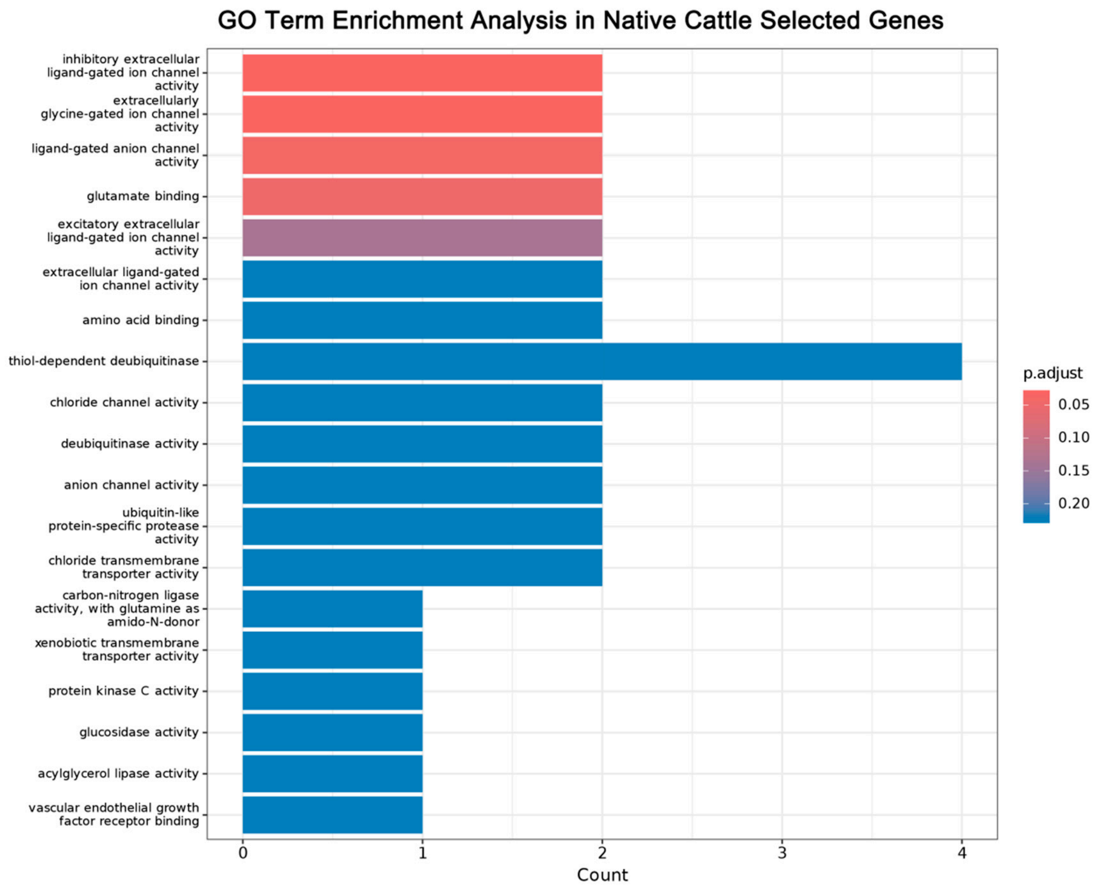


**Figure S4.** GO enrichment analysis of positively selected genes in Chinese indigenous cattle.

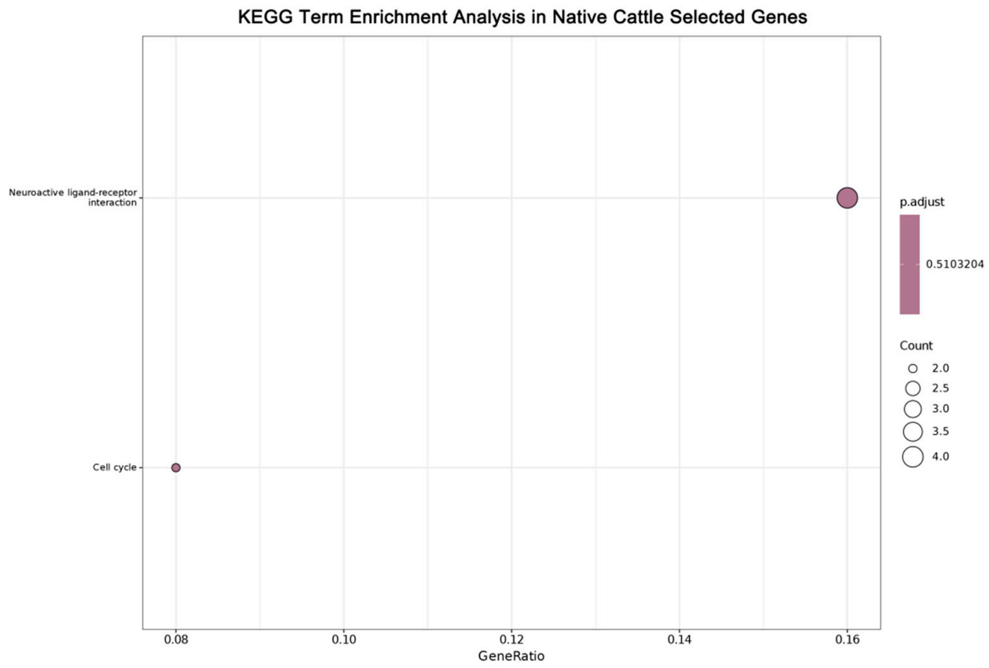


**Figure S5.** KEGG enrichment analysis of positively selected genes in Chinese indigenous cattle.

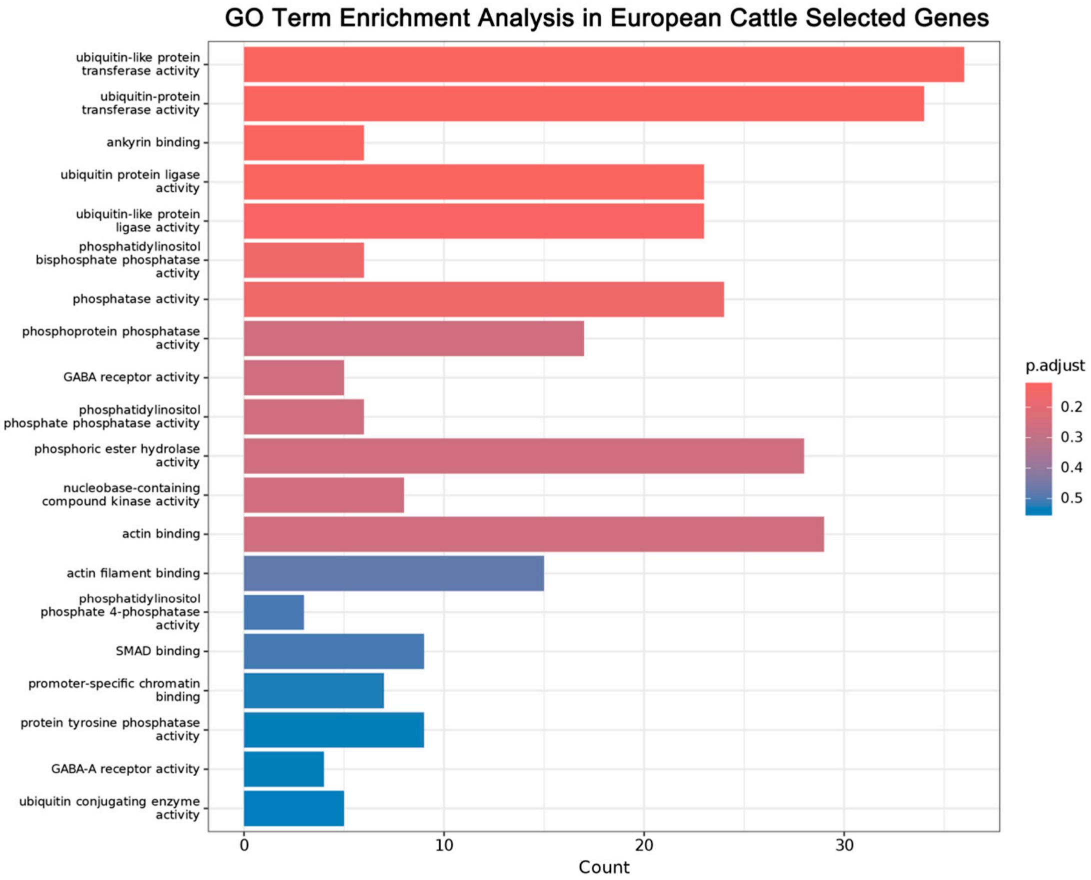


**Figure S6.** GO enrichment analysis of positively selected genes in European cattle.

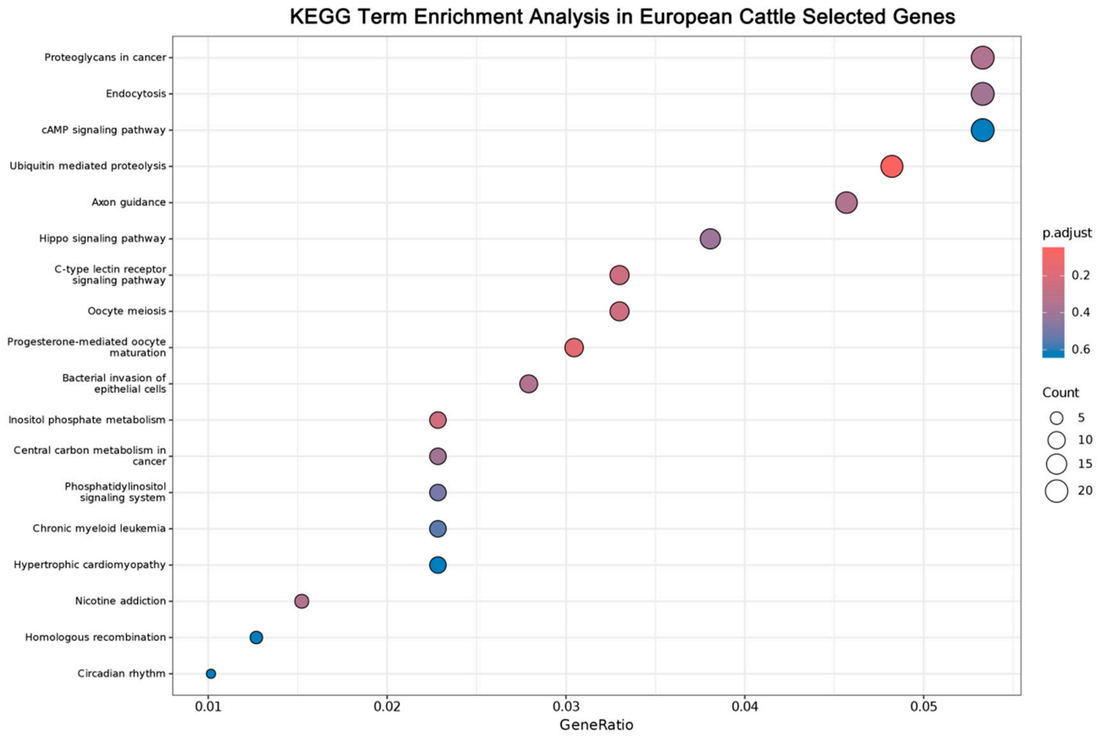


**Figure S7.** KEGG enrichment analysis of positively selected genes in European cattle.
